# Anti-Inflammatory and Antioxidant Activities of Terpene- and Polyphenol-Rich *Premna odorata* Leaves on Alcohol-Inflamed Female Wistar Albino Rat Liver

**DOI:** 10.3390/molecules25143116

**Published:** 2020-07-08

**Authors:** Abeer H. Elmaidomy, Hani A. Alhadrami, Elham Amin, Hanan F. Aly, Asmaa M. Othman, Mostafa E. Rateb, Mona H. Hetta, Usama Ramadan Abdelmohsen, Hossam M. Hassan

**Affiliations:** 1 Department of Pharmacognosy, Faculty of Pharmacy, Beni-Suef University, Beni-Suef 62514, Egypt; abeerabdelhakium@yahoo.com (A.H.E.); elham_bns@yahoo.com (E.A.); mostafa.rateb@uws.ac.uk (M.E.R.); 2 Department of Medical Laboratory Technology, Faculty of Applied Medical Sciences, King Abdulaziz University, P.O. Box 80402 Jeddah 21589, Saudi Arabi; hanialhadrami@kau.edu.sa; 3 Special Infectious Agent Unit, King Fahd Medical Research Centre, King Abdulaziz University, P.O. Box 80402 Jeddah 21589, Saudi Arabia; 4 Department of Medicinal Chemistry and Pharmacognosy, College of Pharmacy, Qassim University, Buraydah 51452, Saudi Arabia; 5 Therapeutic Chemistry Department, National Research Centre, Cairo 11865, Egypt; hanan_abduallah@yahoo.com; 6 Department of Internal Medicine, Faculty of Medicine, Beni-Suef University, Beni-Suef 62514, Egypt; assmaa_med@yahoo.com; 7 School of Computing, Engineering & Physical Sciences, University of the West of Scotland, Paisley PA1 2BE, UK; 8 Department of Pharmacognosy, Faculty of Pharmacy, Fayoum University, Fayoum 63514, Egypt; mhm07@fayoum.edu.eg; 9 Department of Pharmacognosy, Faculty of Pharmacy, Minia University, Minia 61519, Egypt; 10 Department of Pharmacognosy, Faculty of Pharmacy, Deraya University, 7 Universities Zone, New Minia City 61111, Egypt

**Keywords:** *Premna odorata*, LC–HRESIMS, terpene, polyphenolic, ADMET, ROS, TNF-α, antioxidant

## Abstract

*Premna odorata* Blanco (Lamiaceae) is an ethnomedicinal plant native to different tropical regions. Although some reports addressed their anti-inflammatory, cytotoxic, and antituberculotic effects, their hepatoprotective potential is yet to be discovered. Accordingly, this study investigated the crude extract and different fractions of the plant leaves; metabolic profiling using liquid chromatography/high-resolution electrospray ionization mass spectroscopy (LC–HRESIMS) analysis, *in silico* absorption, distribution, metabolism, excretion, and toxicity (ADMET) properties for the dereplicated metabolite via online PreADMET program, ROS scavenger activity on the Hep G2 human liver cancer cell line, and the possible hepatic cellular treatment effects in alcohol-inflamed liver female Wistar albino rats. Metabolic profiling dereplicated a total of 28 metabolites from the crude extract and its various fractions. In silico ADMET and ROS scavenger activity screening suggested plant metabolites are of potential bioactivity. In vivo hepatic treatment with crude, defatted crude, and n-hexane leave extracts suggested all extracts significantly improved liver damage, which was indicated by the reduction of elevated serum levels of bilirubin, AST, ALT, ALP, CRP, TNF-α, ICAM-1, VCAM-1, and MDA. The reduced levels of GSH and TAC were normalized during the study. Histological examinations of liver tissue showed collagen fiber distribution nearly back to its normal pattern. The anti-inflammatory and antioxidant potentials of *Premna odorata* extracts could be partly related to the combined effects of these phytochemicals or their synergistic interactions.

## 1. Introduction

The liver is primarily responsible for alcohol metabolism in the human body and as this is the case, it is vulnerable to alcohol-related injuries [1]. According to literature, chronic alcohol consumption causes liver cell inflammation which triggers an immune response through recognition of damage-associated molecular patterns (DAMPs). DAMPs signal damage or necrosis through transmembrane toll-like receptors (TLRs), a class of proteins that play a key role in the innate immune system [2]. TLRs are single-pass membrane-spanning receptors usually expressed on sentinel cells such as macrophages and dendritic cells and recognized structurally conserved molecules [2,3]. Once recognition occurs, TLRs activate common signaling pathways that activate the nuclear factor kappa-light-chain-enhancer of activated B cells (NF-κB), a key regulator of inflammatory gene expression. This results in the activation of numerous physiological responses: cytokine tumor necrosis factor-α (TNF-α), C-reactive protein (CRP), interlukin-1 (IL-l), IL-6, IL-12, cell adhesion molecules, such as vascular cell adhesion molecule **1** (VCAM-1) and intercellular adhesion molecule **1** (ICAM-1), reactive oxygen species (ROS), inducible nitric oxide synthase (iNOS), and cyclooxygenase II (COX-II) [1,3]. These molecules elicit the production of prostaglandin E_2_ (PGE_2_), chemokines, and various co-stimulatory molecules which play important roles in the pathogenesis of liver cell inflammation [1]. Moreover, the activity of monocytes and neutrophils improve and migrate to inflammation sites and create a cytotoxic environment by releasing noxious chemicals, including ROS, nitrogen species, and various proteinases, which are destructive to both pathogens and host cells. Liquefaction induction of the surrounding hepatic tissue was also observed, resulting in liver cell damage which led to heat, swelling, pain, and loss of tissue function. This is reflected by elevated serum levels of bilirubin, aspartate aminotransferase (AST), alanine aminotransferase (ALT), and alkaline phosphatase (ALP) [1,3].

On the other hand, ROS are naturally produced by living organisms through negative cellular metabolism [4]. At intermediate concentrations, ROS improve physiological cell processes. However, at high ones, ROS adversely modify cell contents as lipid malondialdehyde (MDA), proteins, and deoxyribonucleic acid (DNA) [4]. ROS are classified as free radicals, where superoxide anion (O_2_^−^), hydroxyl radical (OH^−^), and hydrogen peroxide (H_2_O_2_) are considered physiologically significant ROS [4]. The human body naturally contains a variety of antioxidants which counterbalance the effect of ROS such as catalase, glutathione peroxidase (GSH-Px), vitamins (A, C and E), β-carotene, and glutathione (GSH) [4]. As previously indicated, direct liver cell damage that occurs during chronic alcohol consumption is caused by free radicals [5]. In a healthy individual with no liver damage, free radicals are quickly scavenged by natural protective antioxidants (GSH, GSH-Px, vitamins A and E), yet in chronic alcohol consumption individuals, levels of these naturally occurring antioxidants are reduced, GSH being the most affected [5]. Consequently, chronic inflammation progress by ROS could cause organ dysfunction, especially when this process targets important organs like liver [4]. Detecting liver function tests (bilirubin, AST, ALT, and ALP), pro-inflammatory markers (IL-1β, IL-6, IL-10, TNF-α, CRP, nitric oxide (NO), ICAM-1, VCAM-1, and COX-II), oxidative stress marker levels, and antioxidant tests (MDA, H_2_O_2_, GSH-Px, catalase, GSH, and total antioxidant capacity (TAC)) are important steps to determine how much an organ is damaged [1].

Liver disease is a life-threatening disease with elevated mortality rates [6]. Current approaches to treatment, including drug therapy and liver transplantation, feature limited efficacy and risky complications [6]. These concerns have stimulated the search for alternative safe and effective drugs, especially natural drugs, due to their potential in treatment of various forms of hepatopathy [7]. *Premna odorata* is an ethnobotanical plant native to different tropical regions. As a folk medicine, a decoction of its leaves has been used as a febrifuge, diuretic, carminative, and to treat vaginal irritation, abdominal pains, coughing, and dysentery [8]. A limited number of investigations have been carried out on the *Premna* genus, particularly on the *Premna odorata* species, where iridoids, phenylethanoids, flavonoids, and acylated rhamnopyranosides were preliminary described as its main active constituents [9,10,11,12]. Additional studies also addressed their anti-inflammatory, cytotoxic, and anti-tuberculosis effects [9,13,14,15,16]. However, their hepatoprotective potential is yet to be explored. Therefore, this study investigated the crude extract and different fractions of the plant leaves; metabolomics profiling using liquid chromatography/high-resolution electrospray ionization mass spectroscopy (LC–HRESIMS), in silico absorption, distribution, metabolism, excretion, and toxicity (ADMET) properties for the dereplicated metabolites, ROS scavenger activity, and the possible hepatic cellular treatment effects in alcohol-inflamed liver female Wistar albino rats.

## 2. Results

### 2.1. Metabolomic Analysis

**Figure 1:**
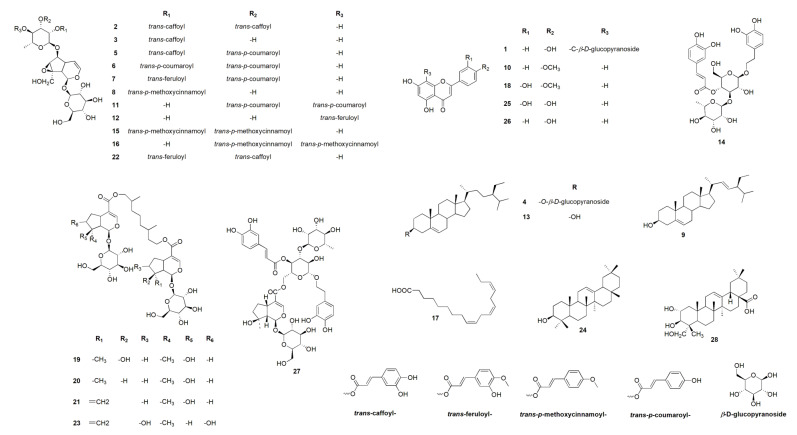
Dereplicated metabolites from the LC–HRESIMS analysis of the crude *Premna odorata* leave extract and fractions.

Chemical profiling of the secondary metabolites of *Premna odorata* leaves using LC–HRESIMS for dereplication purposes resulted in the characterization of a variety of constituents, including sterols, triterpenes, fatty acids, iridoids, flavones, and phenylethanoids (Table 1, Figure 1). From the metabolomics data, the ion at mass-to-charge ratio *(m/z*) 433.1361 corresponding to the suggested molecular formula C_21_H_20_O_10_ was dereplicated as vitexin (**1**) [9,17], which was formerly reported in *Premna odorata*. Two acylated iridoid glycosides with the molecular formula C_39_H_44_O_20_ and C_30_H_38_O_17_ were characterized as premnoside A (**2**) and 6-*O*-α-L-(2’’-O-trans-caffoyl) rhamnopyranosyl catalpol (**3**) from the ions at *m/z* 833.2746 and 671.1910, respectively, which were previously reported in *Premna odorata* [11,12]. Furthermore, the ion at *m/z* 577.1969 with the corresponding predicted molecular formula C_35_H_60_O_6_ was dereplicated as the steroidal glycoside daucosterol (**4**), which was earlier isolated from *Premna japonica*, whereas herein, it is reported from the *Premna odorata* plant for the first time [18]. Additional sterols, triterpenes, fatty acids, iridoids, phenylethanoids, and flavone-related compounds which were formally isolated from *Premna odorata* leaves were also characterized as compounds **5**–**7**, **9**–**11**, **13**–**16**, **18**, **19**–**22**, and **24**–**26** based on the ions and the corresponding predicted molecular formulas (Table 1, Figure 1) [8,9,10,11,19]. The ions at *m/z* 669.1634 and 685.2780 with the predicted molecular formulas C_31_H_40_O_16_ and C_31_H_40_O_17_ were dereplicated as the iridoid glycosides 6-*O*-α-L-(2’’-*O*-trans-p-methoxycinnamoyl) rhamnopyranosyl catalpol (**8)** and 6-*O*-α-L-(4’’-*O*-trans-feruloyl) rhamnopyranosyl catalpol (**12**) [18], which were first reported in the *Premna* genus. Likewise, iridoids with the molecular formula C_42_H_64_O_20_ and C_45_H_58_O_24_ were identified as premnaodoroside D (**23**) [20] and premcoryoside (**27**) [21], which were reported for the first time in the *Premna* genus. Furthermore, the ions at *m/z* 277.1807, and 489.2793 with the suggested molecular formulas C_18_H_30_O_2_ and C_30_H_48_O_5_ were identified as the unsaturated fatty acid linolenic acid and the pentacyclic triterpene arjunolic acid (**13** and **28**, respectively) [22]. It is worth mentioning that based on dereplication, sterols, triterpenes, and the fatty acid were predominantly in the n-hexane (n-hex) fraction, while iridoids, flavones, and phenylethanoids were the major metabolites in the dichloromethane (DCM), ethyl acetate (EtOAC), and n-butanol fractions (Table 1, Figure 1).

### 2.2. In Silico ADMET Properties for the Crude Extract of Different Premna odorata Metabolites

In silico predicted ADMET profiling of the dereplicated secondary metabolites using PreADMET program version 2.0 showed that flavones (vitexin (**1**), acacetin (**10**), diosmetin (**18**), luteolin, and apigenin (**25**–**26**) had low to middle absorption to the blood–brain barrier (BBB), moderate to high human intestinal absorption (HIA), weak to strong plasma protein binding (PPB), moderate skin permeability (SP), middle permeability to heterogeneous human epithelial colorectal adenocarcinoma Caco-2 cells and the Madin–Darby Canine Kidney (MDCK) cell model. Additionally, all flavones inhibited cytochrome P_450_-2C_19_ (CYP_2_C_19_), CYP_2_C_9_, and CYP_3_A_4_ with no effect on the permeability glycoprotein (Pgp) (Table 2). Toxicity screening results using PreADMET for flavone aglycones showed mutagenicity using the Ames test except for **1**. Moreover, flavones showed potential rat and rodent carcinogenicity, except for **1**, which showed carcinogenicity in rats only. Furthermore, the human Ether-à-go-go-Related Gene (hERG) inhibition is of moderate to high risk (Table 3). Moreover; iridoids (premnoside A and 6-*O*-α-L-(2’’-*O*-trans-caffoyl) rhamnopyranosyl catalpol (**2**–**3**), premnoside C, D, H, and 6-*O*-α-L-(2’’-*O*-trans-methoxycinnamoyl) rhamnopyranosyl catalpol (**5**–**8**), premnoside G and 6-*O*-α-L-(4’’-*O*-trans-feruolyl) rhamnopyranosyl catalpol (**11**–**12**), premnoside E and F (**15**–**16**), premnaodoroside A–D and premnoside D (**19**–**23**), premcoryoside **27**) and phenylethanoid verbascoside (**14**) had low absorption to the BBB, except for compounds **11**, **15**, and **16** which showed moderate absorption, poor HIA, except for compounds **11**, **15**, and **16** which showed moderate results, weak PPB, poor SP, middle Caco-2, MDCK permeability. Furthermore, all iridoids inhibited Pgp, CYP_2_C_19_, CYP_2_C_9_, and CYP_3_A_4_ (Table 2). Toxicity screening results using PreADMET for iridoids and phenylethanoids showed no mutagenicity using the Ames test, except for compounds **19**, **21**, and **23** which showed positive mutagenicity with the TA100-NA strain and negative mutagenicity with the other strains used. Moreover, iridoids and phenylethanoids showed no potential rat carcinogenicity except against rodents. Also, hERG inhibition was ambiguous, except for compounds **8** and **15**–**16**, which were of high risk (Table 3). The sterols, triterpenes, and fatty acids (daucosterol (**4**), stigmasterol (**9**), β-sitosterol (**13**), linolenic acid (**17**), β-amyrin (**24**), arjunolic acid (**28**) had middle to strong absorption to the BBB, high HIA, strong PPB, good SP, middle Caco-2, MDCK permeability; furthermore, all of them inhibited Pgp, CYP_2_C_9_, and CYP_3_A_4_ (Table 2). Toxicity screening results showed no mutagenicity using the Ames test, except for compound **17**. Moreover, these compounds showed potential rat and rodent carcinogenicity, except for compounds **4** and **13**, which showed carcinogenicity against rodents only. Furthermore, hERG inhibition is of low to moderate risk (Table 3). 

HERG: human Ether-à-go-go-Related Gene. The Ames test is a simple method of testing mutagenicity of a compound. PreADMET predicts toxicity for TA98, TA100, and TA1535, which are often used in the Ames test. The prediction result is positive or negative. Rodent carcinogenicity is a toxicity that causes cancer in the body. PreADMET predicts the result from its model, which is built upon the data of the National Toxicology Program (NTP) and the United States Food and Drug Administration (US FDA), which are the results of the 2-year-long in vivo carcinogenicity tests of mice and rats. Negative prediction indicates clear evidence of carcinogenic activity, and positive prediction shows no evidence of carcinogenic activity. The hERG is a gene that codes for a protein known as K_v_11.1, the alpha subunit of a potassium ion channel. This ion channel is best known for its contribution to the electrical activity of the heart. The hERG channel mediates the repolarizing I_Kr_ current in the cardiac action potential, which helps coordinate the heart’s beating.

### 2.3. In Vitro Measurement of Total Reactive Oxygen Species (ROS)

**Figure 2:**
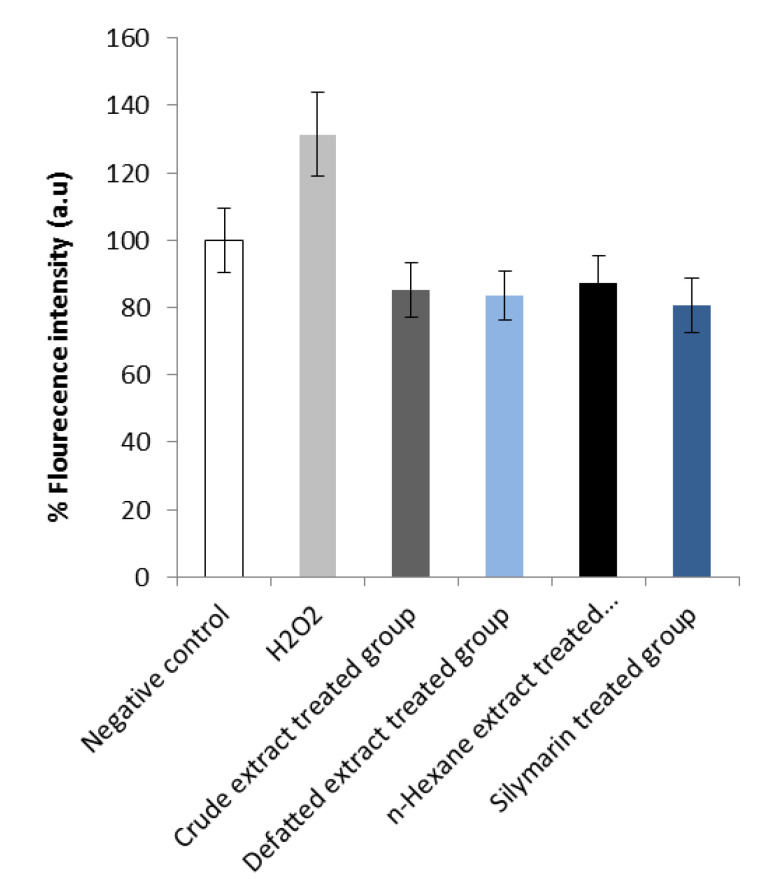
Reactive oxygen species (ROS) induction by H_2_O_2_ in the Hep G2 human liver cancer cell line: scavenging effects of various *Premna odorata* extracts. The cells were treated with 100 µL and evaluated for ROS production as described in the Materials and Methods. The data (means ± SD) are representative of three independent experiments. Significant difference at *p* ˂ 0.05 versus H_2_O_2_.

Various *Premna odorata* extracts (crude, defatted crude, and n-hex) were screened for their ability to scavenge the ROS in the Hep G2 human liver cancer cell line at 100 μL using the Reactive Oxygen Species Assay (Beyotime Institute of Biotechnology) (Figure 2). The results showed that the treatment with all the extracts significantly decreases the Hep G2 ROS content, reflecting activities of their antioxidants. Silymarin with defined antioxidant activity was used as a standard reference drug at a 100 μL dose.

### 2.4. Acute Toxicity

In this study, the acute toxicity test of the crude, defatted crude, and n-hex extracts showed no signs of toxicity up to 5 g/kg body weight (b.w.); they were considered safe. Therefore, all extracts were investigated at a dose of 1/10 (500 mg/kg b.w.) in the alcohol-inflamed liver of female Wistar albino rats.

### 2.5. Potential Effects of Premna odorata Extracts in Liver Function Tests

In response to the alcohol-inflamed liver, bilirubin, AST, ALT, and ALP showed a significant increase in their serum levels with the percentage reaching 65.88%, 108.53%, 33.05%, and 34.04%, respectively, when compared to the negative control group (Table 4). Treatment of alcohol-inflamed liver rats with crude, defatted crude, n-hex extracts showed a significant decrease in the bilirubin, AST, ALT, and ALP levels in all treated groups when compared to the silymarin positive control group. The crude leave extract showed the highest percentage of improvement for AST (143.23%) and ALP (48.47%). While bilirubin showed a significant improvement upon treatment with crude and n-hex extracts (58.11%), the defatted crude extract showed the best improvement of the ALT level (66.68%) in all treated groups (Table 4).

### 2.6. Potential Effects of Premna odorata Extracts on Oxidative Stress Markers, and Antioxidant

In response to the alcohol-inflamed liver state, the MDA level showed a significant increase (42.97%) when compared to the negative control group. The GSH and TAC had a significant decrease of 27.42% and 38.46%, respectively (Table 4). Treatment of alcohol-inflamed liver rats with crude, defatted crude, n-hex extracts showed a significant increase in the GSH and TAC levels in all treated groups compared to the silymarin positive control group (Table 4). Defatted crude and n-hex extracts showed the highest percentage of improvement for the GSH (50.32%) and TAC (42.30%). MDA showed a significant decrease in all treated groups, whereas the crude extract showed the best amelioration −71.07% (Table 4).

### 2.7. Potential Effects of Premna odorata Extracts on Inflammatory Markers and Adhesion Molecules

In response to the alcohol-inflamed liver state, CRP, TNF-α, ICAM-1, and VCAM-1 parameters showed a significant increase in their levels, 86.96%, 98.49%, 114.54%, and 51.72%, respectively, when compared to the negative control group (Table 4). Treatment of alcohol-inflamed liver rats with crude, defatted crude, n-hex extracts showed a significant decrease in CRP, TNF-α, VCAM-1, and ICAM-1 when compared to the silymarin positive control group (Table 2). The n-hex fraction recorded the highest percentage of improvement for CRP (86.20%) and ICAM-1 (96.36%). The defatted crude extract showed the highest percentage of amelioration for TNF-α (102.56%). VCAM-1 was improved upon treatment with defatted crude and n-hex extracts (41.37%) (Table 4).

### 2.8. Histopathological Investigation of Liver

**Figure 3:**
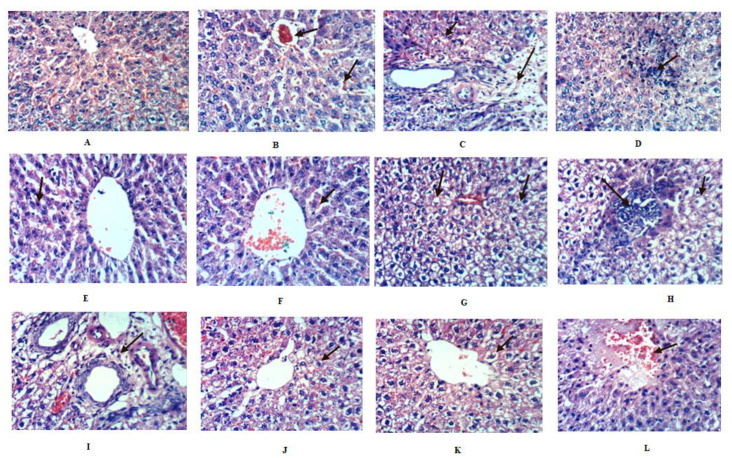
Histopathological results of the activity for *Premna odorata* extracts (crude, defatted, and n-hexane) and silymarin in alcohol-inflamed liver female Wistar albino rats using a dose of 500 and 200 mg/kg b.w., respectively (H and E X 400). (**A**) The liver of a rat from group 1 (negative control) showing the negative histological structure of a hepatic lobule. (**B**) The liver of a rat from group 2 (positive control) showing congestion of the central vein and hepatic sinusoids. (**C**) The liver of a rat from group 2 showing cytoplasmic vacuolization of hepatocytes and a portal edema. (**D**) The liver of a rat from group 2 showing focal hepatic necrosis associated with inflammatory cell infiltration. (**E**) The liver of a rat from group 3 (crude extract-treated group) showing necrosis of sporadic hepatocytes. (**F**) The liver of a rat from group 3 showing slight congestion of hepatic sinusoids. (**G**) The liver of a rat from group 4 (defatted extract-treated group) showing slightly hydropic degeneration of hepatocytes. (**H**) The liver of a rat from group 4 showing hydropic degeneration of hepatocytes and focal hepatic necrosis associated with inflammatory cell infiltration. (**I**) The liver of a rat from group 5 (n-hexane-treated group) showing a slightly portal edema. (**J**) The liver of a rat from group 5 showing slightly hydropic degeneration of hepatocytes. (**K**) The liver of a rat from group 6 (silymarin-treated group) showing slightly hydropic degeneration of hepatocytes. (**L**) The liver of a rat from group 6 showing slightly focal hepatic hemorrhage.

The histological investigation of liver sections supported the biochemical results (Figure 3). Liver sections for the control rat group showed normal lobular architecture and normal hepatic cells with a well-preserved cytoplasm and a well-defined nucleus (Figure 3A). Alcohol-inflamed liver (positive control) showed a necrobiotic change of the hepatocytes, including congestion of the central vein and hepatic sinusoids, cytoplasmic vacuolization of hepatocytes, portal edema, and focal hepatic necrosis associated with inflammatory cell infiltration (Figure 3B–D). Treatment of alcohol-inflamed liver rats with *Premna odorata* extracts (crude, defatted crude, and n-hex) (Figure 3E–I) showed positive results for all extracts in which focal tubular necrosis associated with inflammatory cell infiltration was minimum, where the crude extract was the most active followed by the defatted crude and the n-hex extracts. The slight congestion of hepatic sinusoids was also observed. The silymarin-treated group (Figure 3K–L) showed a slightly hydropic regeneration of hepatocytes and focal hepatic hemorrhage.

## 3. Discussion

Metabolic profiling of *Premna odorata* dereplicated 28 metabolites from the various fractions of the crude leave extract. The identified metabolites belonged to various chemical classes, including iridoids (monoterpenes), triterpenes, flavones, and phenylethanoids. According to literature, pharmacokinetic parameters of compounds, such as absorption, distribution, metabolism, excretion, and toxicity (ADMET), are important in order to determine the potential success of a compound for therapeutic use. Some important chemical descriptors correlate well with ADMET properties, for instance, HIA as a primary determinant of oral absorption of a fraction. The distribution of each compound in the human body depends on many factors, such as permeability of the BBB, Caco-2, MDCK, SP, and PPB. Similarly, the metabolism and excretion of most drugs also depend on many factors, like CYP_2_C_19_, CYP_2_C_9_, CYP_3_A_4_, and Pgp. In silico predicted ADMET profiling of the dereplicated secondary metabolites present in *Premna odorata* leaves showed that flavones (vitexin (**1**), acacetin (**10**), diosmetin (**18**), luteolin, and apigenin (**25**–**26**) had low to middle absorption to the BBB, moderate to high HIA, weak to strong PPB, moderate SP, and middle Caco-2 and MDCK permeability. Furthermore, all flavones inhibited CYP_2_C_19_, CYP_2_C_9_, and CYP_3_A_4_ and had no significant effect on Pgp (Table 2). Toxicity screening results from PreADMET for the flavone aglycones showed mutagenicity using the Ames test, except for **1**. Moreover, all flavones showed potential rat and rodent carcinogenicity, except for **1**, which only showed carcinogenicity against rats. Furthermore, a moderate to high risk of hERG inhibition was predicted (Table 3). The iridoids (premnoside A and 6-*O*-α-L-(2’’-*O*-trans-caffoyl) rhamnopyranosyl catalpol (**2**–**3**), premnoside C, D, H, and 6-*O*-α-L-(2’’-*O*-trans-methoxycinnamoyl) rhamnopyranosyl catalpol (**5**–**8**), premnoside G and 6-*O*-α-L-(4’’-*O*-trans-feruolyl) rhamnopyranosyl catalpol (**11**–**12**), premnoside E, F (**15**–**16**), premnaodoroside A–D and premnoside D (**19**–**23**), premcoryoside (**27**) and phenylethanoid verbascoside (**14**) had low absorption to the BBB, except for **11**, **15**, and **16**, which showed moderate absorption, poor HIA, except for **11**, **15**, **16**, which showed moderate results, weak PPB, poor SP, and moderate Caco-2 and MDCK permeability. Furthermore, all iridoids inhibited Pgp, CYP_2_C_19_, CYP_2_C_9_, and CYP_3_A_4_ (Table 2). Toxicity screening results from PreADMET for the iridoids showed no mutagenicity using the Ames mutagenicity test, except for **19**, **21**, and **23**, which showed positive mutagenicity for the TA100-NA strain and negative mutagenicity for the other strains used. Moreover, iridoids and phenylethanoids showed no potential rat carcinogenicity except against rodents. Furthermore, hERG inhibition was ambiguous, except for **8**, and **15**–**16** were of high risk (Table 3). The sterols, triterpenes, and fatty acids (daucosterol (**4**), stigmasterol (**9**), β-sitosterol (**13**), linolenic acid (**17**), β-amyrin (**24**), arjunolic acid (**28**)) had moderate to strong absorption to the BBB, high HIA, strong PPB, good SP, middle Caco-2, MDCK permeability, and all inhibited Pgp, CYP_2_C_9_, and CYP_3_A_4_ (Table 2). Toxicity screening results showed no mutagenicity using the Ames mutagenicity test, except for **17**. Moreover, these compounds showed potential rat and rodent carcinogenicity, except for **4** and **13**, which showed mutagenicity against rodents only. Furthermore, hERG inhibition is of low to moderate risk (Table 3). The results of in silico screening highlight that *Premna odorata* metabolites theoretically are potentially highly bioactive. These features are reflected by their low plasma concentrations due to their extensive cellular uptake [24]. Tight regulation of the compound plasma concentrations was accomplished through binding to serum albumin [25], which lowers plasma concentrations of unbound bioactive compounds, while offering a storage system for delayed release into the plasma. In addition, albumin-bound compounds are more stable against oxygen-dependent degradation, resulting in prolongation of their biological availability and further extension of their plasma’s half-life. This response would protect target tissues from high-level uptake of compounds, which might trigger a toxic reaction in cells [25]. Indeed, prolonged release of these compounds from plasma proteins would ensure a more constant rate of cellular uptake [26]. On the other hand, the ADME properties of these compounds increased their toxicity and carcinogenicity. Generally, higher lipophilicity of compounds leads to increased metabolism and poor absorption along with an increased probability of binding to undesired hydrophobic macromolecules, thereby increasing the potential for toxicity [27] (Table 2 and Table 3).

Literature indicated there is a relationship between liver disease and heavy alcohol consumption, as liver is the primary site of alcohol metabolism [28]. There are two pathways for alcohol metabolism in the liver [29]. The first is the alcohol dehydrogenase (ADH) pathway, which converts alcohol through oxidation processes to acetaldehyde which was found to be highly toxic to the body even in low concentrations. The second, the microsomal enzyme oxidizing system (MEOS) pathway that oxidizes alcohol to acetaldehyde by cytochrome P_450_2E_1_, or CYP_2_E_1_. In the latter, activation increases ROS production. Unfortunately, an increase in alcohol consumption also activates the MEOS pathway, resulting in the increased ROS production [29]. Normally, ROS are quickly scavenged by natural protective antioxidants (GSH, GSH-Px, vitamins A and E), but chronic alcohol consumption diminishes antioxidant levels and renders liver cells more susceptible to free radical-induced injuries [5]. This destructive interaction with vital cell constituents potentially causes cell death, resulting in the sequential degradation of cell membranes by a lipid peroxidation process [5]. Consequently, chronic alcohol consumption causes severe liver cell inflammation [5], which results in activation of the NF-κB pathway, increasing the production of TNF-α, CRP, IL-l, IL-6 and IL-12, VCAM-1 and ICAM-1, ROS, iNOS, and COX-II [1,3]. These molecules together elicit the production of PGE_2_, chemokines, and various co-stimulatory molecules, which play important roles in the pathogenesis of liver cell inflammation [1].

Metabolic profiling of the crude *Premna odorata* leave extract dereplicated various anti-inflammatory and antioxidants compounds. Vitexin (**1**) showed significant inhibition to TNF-α and NO [30], acacetin (**10**) and apigenin (**26**) inhibited expression of ICAM-1, VCAM-1, and selective inhibition of prostaglandin synthesis and IL-6, 8 production [23,31]. Diosmetin (**10**) prevented the generation of intracellular ROS and the formation of MDA, increased the effects of the intracellular antioxidant enzymes superoxide dismutase (SOD), catalse (CAT), and GH-Px, and suppressed the iNOS activity [32]. Diosmetin also exerted an anti-inflammatory effect by reducing NO production and TNF-α release, reduced the enzyme activities of COX-II and PGE_2_, and blocked the NF-κB signaling pathway [32]. Luteolin (25) also blocked the NF-κB signaling pathway and VCAM-1 expression [33]. Moreover, the crude *Premna odorata* leave extract contained iridoids which mainly acylated with aromatic acids (coumaric, P-methoxy cinnamic, caffeic, or ferulic) (Table 1, Figure 1), and aliphatic dimer iridoids (**2**–**3**, **5**–**8**, **11**–**12**, **15**–**16**, **19**–**23**, **27**), both of which prevented the generation of ROS and MDA, increased the activity of SOD, CAT, and GH-Px, and suppressed the iNOS activity. Furthermore, they reduced TNF-α release and the enzyme activities of COX-II and PGE_2_ and blocked the NF-κB signaling pathway [34,35]. Furthermore, the crude *Premna odorata* extract contained daucosterol (**4**), stigmasterol (**9**), β-sitosterol (**13**), linolenic acid (**17**), β-amyrin (**24**), and arjunolic acid (**28**) and was reported to block the NF-κB signaling pathway [36,37,38] (Table 1, Figure 1). The reported data, ADMET, and ROS antioxidant scavenger activities against the content of various *Premna odorata* extracts (Figure 2) encourage further in-depth investigation of the plant extract’s effect on an inflamed liver. In this study, the hepatic treatment potential of the crude extract of *Premna odorata* leaves and its fractions (crude, defatted total, n-hex) were investigated on the alcohol-inflamed liver of female Wistar albino rats. The different fractions were chosen according to the obtained crude extract weight 200g/3kg dried leaves and its different fractions (n-hexane, dichloromethane, ethyl acetate, and n-butanol, 20, 3, 5, 70 g/3kg dried leaves, respectively) and focusing on the distribution of iridoids, flavones, and polyphenolic metabolites according to the LC–HRESIMS profiling (Table 1, Figure 1).

In response to the alcohol-inflamed liver, bilirubin, AST, ALT, and ALP levels showed a significant increase in their levels, 65.88%, 108.53%, 33.05%, and 34.04%, respectively, when compared to the negative control group (Table 4). The MDA level showed a significant increase (42.97%), while the GSH and TAC recorded a significant decrease of 27.42% and 38.46%, respectively (Table 4). Furthermore, CRP, TNF-α, ICAM-1, and VCAM-1 parameters showed a significant increase in their levels, 86.96%, 98.49%, 114.54%, and 51.72%, respectively, compared to the negative control group (Table 4). Treatment of alcohol-inflamed liver rats with crude, defatted, n-hex extracts showed a significant decrease in bilirubin, AST, ALT, ALP, MDA, CRP, TNF-α, VCAM-1, and ICAM levels, as compared to the silymarin-positive control group in all treated groups (Table 4) accompanied by a significant increase in the GSH and TAC levels (Table 4). The crude extract showed the highest percentage of improvement for AST (143.23%), ALP (48.47%), and MDA (71.07%). Bilirubin was equally improved upon treatment with crude and n-hex extracts (58.11%). On the other hand, the defatted extract showed the highest percentage of improvement for ALT (66.68%), GSH (50.32%), and TNF-α (78.18%) (Table 4). The n-hex extract showed the highest percentage of improvement for the TAC (42.30%), CRP (86.20%), and ICAM-1 (96.36%). VCAM-1 showed the highest percentage of improvement with defatted and n-hex extracts (41.37%). These biochemical findings were also simultaneously substantiated with the histopathological observations described before (Figure 3).

In response to the biochemical parameters above and according to the LC–HRESIMS metabolomic profiling, the n-hex fraction contained sterols, triterpenes, and fatty acids (Table 1, Figure 1). These metabolites have been shown to interfere mainly with the expression of pro-inflammatory cytokines and adhesion molecules by blocking the NF-κB activity [36,37,38]. We posit this explains the significant effect of the n-hex extract in reducing the serum levels of CRP, TNF-α, VCAM-1, and ICAM (Table 4). The defatted extract mainly contained acylated iridoids, flavones, and phenylethanoids, which were mainly acylated with coumaric, P-methoxy cinnamic, or ferulic aromatic acids (Table 1, Figure 1). Having surveyed the relevant literature, defatted extract metabolites were also found to interfere with the expression of pro-inflammatory cytokines and interleukins by blocking the NF-κB activity (especially TNF-α), their activity increasing with the number of free phenolic (-OH) groups [8,34,35]. This phenomenon was observed through the increased TNF-α improving level (102.56%) for the defatted extract compared to other treated groups (Table 4). Additionally, the various extracts of *Premna odorata* showed potential anti-inflammatory activity which improved the severe liver cell inflammation accompanied by chronic alcohol consumption (Table 4).

The significant reduction of MDA levels and enhancement of the TAC and GSH (Table 4) in all treated groups reflected the antioxidant activity for *Premna odorata* metabolites, especially verbascoside (**14**). The latter has been reported as an inhibitor of P_450_2E_1_ or CYP_2_E_1_, therefore, blocking the MEOS metabolic pathway besides having free radical scavenging effects [39]. The net results showed the improvement of endogenous scavenging of free radicals and the total antioxidant potential, which preserves the structural integrity of hepatocytes [7]. As a result of the anti-inflammatory and antioxidant activities of the *Premna odorata* extracts, the elevated levels of ALT and AST were markedly reduced, thus suggesting the stabilization of plasma membranes in addition to repairing the hepatocellular damage. Moreover, the decrease of the raised ALP and bilirubin levels indicated the improvement of the biliary dysfunction (Table 4) [7].

According to literature, hepatic treatment investigations were conducted on other *Premna* species, including the study of hepatoprotection of alcoholic *Premna esculenta**,** Premna corymbosa, Premna serratifolia*, and *Premna tomentosa* leave extracts after liver induction with CCl_4_. These studies showed significant hepatoprotection by decreasing the activity of serum enzymes, bilirubin, and lipid peroxidation [40,41,42,43]. Moreover, the hepatoprotective role of the ethanol *Premna integrifolia* leave extract on the aflatoxin B_1_-induced toxicity was studied in mice; the study showed the protective effect of *Premna integrifolia* through the restoration of altered hematological indices and liver marker enzymes [44]. The *Premna* genus is a natural source for bioactive metabolites having anti-inflammatory and antioxidant properties which have a direct effect on liver inflammation.

## 4. Materials and Methods

### 4.1. Chemicals

The solvents used in this work were n-hexane (n-hex.; boiling point (b.p.) 60–80 ˚C), dichloromethane (DCM), ethyl acetate (EtOAC), n-butanol (n-but.), ethanol (EtOH), and H_2_O_2_ purchased from El-Nasr Company for Pharmaceuticals and Chemicals, Egypt. All chemicals for kits (Table 5) were of a high analytical grade and purchased from Sigma Chemical Co Ltd. (St Louis, MO, USA). All the kits were produced by Biosystems SA Costa Brava 30, Barcelona (Spain), and DiaSys Diagnostic Systems GmbH, Germany.

### 4.2. Plant Material and Extraction

*Premna odorata* leaves were collected at the flowering stage in May 2018 from Zoo, Giza, Egypt. The plant was identified by Abd El-Halim A. Mohammed (Horticultural Research Institute, department of Flora and phytotaxonomy Researchers, Dokki, Cairo, Egypt). A voucher specimen (2018-BuPD 45) was deposited at the department of pharmacognosy, Faculty of Pharmacy, Beni-Suef University, Egypt.

The air-dried leaves (3 kg) were collected and air-dried in the darkness for one month. After drying, the leaves were finely powdered using a CM 290 Cemotec™ laboratory grinder (200–230 V, 50–60 Hz, Foss, Denmark). The finely powdered leaves were extracted by maceration without agitation using 70% ethanol (EtOH), (4 L, 3 X, four days each) at room temperature and subsequently concentrated under vacuum at 40 °C using a rotary evaporator (Buchi Rotavapor R-300, Cole–Parmer, Vernon Hills, IL, USA) to afford a 200 g crude extract. A 20 g aliquot was used for all biological evaluations of the crude extract. Another 2 g crude extract was suspended in 4 mL distilled water and successively Kuching-partitioned with solvents of different polarities (n-hex, DCM, EtOAc, and n-but.) for the LC–HRESIMS experiment. The remaining 178 g crude extract was defatted using n-hex (1 L, 3 h, 3 X). The n-hexane extract was dried at 30 °C using a rotary evaporator (Buchi Rotavapor R-300, Cole–Parmer, Vernon Hills, IL, USA) to obtain the 20 g used for all biological evaluations of the n-hex extract. The remaining extract now defatted by n-hex was dried and 20 g were used as the defatted total extract.

### 4.3. Metabolomic Analysis Procedure

The crude extract of *Premna odorata* leaves and the various fractions (n-hexane, DCM, EtOAc, and n-butanol) were subjected to metabolomic analysis using LC–HRESIMS [45,46]. LC–HRESIMS metabolic analyses were done using an ACQUITY Ultra Performance Liquid Chromatography system coupled with a Synapt G_2_-HDMS quadrupole-time-of-flight hybrid mass spectrometer (Waters, Milford, MA, USA). Chromatographic separation was carried out on an ethylene bridged hybrid (BEH) C_18_ column (2.1 × 100 mm, particle size 1.7 µm; Waters, Milford, MA, USA) with a guard column (2.1 × 5 mm, particle size 1.7 µm) and a linear binary solvent gradient of 0–100% eluent B over 6 min at a flow rate of 0.3 mL/min^−1^ using 0.1% formic acid in water (v/v) as solvent A and acetonitrile as solvent B. The injection volume was 2 µL and the column temperature was 40 °C. Electrospray ionization (ESI) in the positive mode was used and the source was operated at 120 °C. The ESI capillary voltage was set to 0.8 kV, the sampling cone voltage was set to 25 V, and nitrogen (at 350 °C, flow rate (FR) 800 L/h) was used as the desolvation gas and the cone gas (FR 30 L/h). The mass range for the TOF–MS was set according to the mass-to-charge ratio (*m/z*) 50–1,200. In MZmine 2.12, the raw data were imported by selecting the ProteoWizard-converted positive files in the mzML format. Ions were detected, followed by a chromatogram builder and a chromatogram deconvolution. The local minimum search algorithm was applied, and isotopes were also identified via the isotopic peaks grouper. The missing peaks were detected using a gap-filling peak finder. An adduct search as well as a complex search were performed. The processed data set was then subjected to molecular formula prediction and peak identification. The positive data set from each of the respective plant extracts were dereplicated against the Dictionary of Natural Products (DNP) database.

### 4.4. In Silico ADMET Properties for the Crude Extract of Different Premna odorata Metabolites

In silico ADMET properties for the metabolites identified by the Premna odorata metabolomics profiling using LC–HRESIMS were defined using the online PreADMET program version 2.0 depending on the 2D structural models drawn in the ChemBioDraw Ultra version 11.0 software (Cambridge Software), including plasma protein binding (PPB), where the drug is considered; chemicals are strongly PPB at ˃ 90% and weakly at ˂ 90%. The blood–brain barrier (BBB); the drug is considered to have high absorption to the CNS ˃ 2.0 BB (Cbrain/Cblood), moderate absorption to the CNS at 2.0~0.1 BB (Cbrain/Cblood), and low absorption to the CNS at ˂ 0.1 BB (Cbrain/Cblood) [47]. For skin permeability (SP), PreADMET predicts in vitro skin permeability and the result value is given as logKp. Kp (cm/h) is defined as follows: KP =$\;\frac{\mathrm{km}\times D}{h}$*,* where *Km* is the coefficient of distribution between the stratum corneum and the vehicle, *D* is the average diffusion coefficient (cm^2^/h), and *h* is the thickness of skin (cm) [48]. In human intestinal absorption (HIA) [49], the drug is considered a poorly absorbed compound at 0~20% HIA, a moderately absorbed compound at 20~70% HIA, and a well-absorbed compound at 70~100% HIA. In the Caco-2 cell permeability and Madin–Darby canine kidney (MDCK) cell model [50,51], the drug could generally belong to one of three categories: low permeability ˂ 4 P_Caco−2_ (nm/sec), moderate permeability 4~70 P_Caco−2_ (nm/sec), and high permeability ˃ 70 P_Caco−2_ (nm/sec). Permeability glycoprotein (Pgp) is an important protein of the cell membrane that pumps many foreign substances out of cells, and it likely evolved as a defense mechanism against harmful substances [52]. Cytochromes P_450_-2C_19_ (CYP_2_C_19_), CYP_2_C_9_, and CYP_3_A_4_ are important cytochrome P_450_ enzymes with a major role in the oxidation of both xenobiotic and endogenous compounds [53]. The Ames test is a simple method of testing mutagenicity of a compound [54]. PreADMET predicts toxicity for TA98, TA100, and TA1535, which are often used in the Ames test. The prediction result is positive or negative. Rodent carcinogenicity is a toxicity that causes cancer in the body. PreADMET predicts the result from its model, which is built upon the data of the National Toxicology Program (NTP) and the United States Food and Drug Administration (US FDA), which are the results of the 2-year-long in vivo carcinogenicity tests of mice and rats. Negative prediction indicates clear evidence of carcinogenic activity and positive prediction shows no evidence of carcinogenic activity.

The hERG (the human Ether-à-go-go-Related Gene) is a gene that codes for a protein known as K_v_11.1, the alpha subunit of a potassium ion channel. This ion channel is best known for its contribution to the electrical activity of the heart. The hERG channel mediates the repolarizing I_Kr_ current in the cardiac action potential, which helps coordinate the heart’s beating [55].

### 4.5. In Vitro Measurement of Total Reactive Oxygen Species (ROS)

#### 4.5.1. Cell Lines, Culture Conditions

The Hep G2 human liver cancer cell line was obtained from the American Type Culture Collection, the cells were cultured using Roswell Park Memorial Institute (RPMI 1640 medium supplemented with 10% fetal calf serum, 100 U/mL penicillin, and l00 μg/mL streptomycin) in a humidified atmosphere with 7% CO_2_ and 93% air at 37 °C.

#### 4.5.2. Intracellular ROS Levels Quantification

ROS inhibition activities of crude, defatted crude, and n-hex *Premna odorata* extracts were tested against the Hep G2 human liver cancer cell line using the ROS flow cytometry assay [56]. In brief, the cells were placed in a 96-well microtiter plate at a density of 1 × 10^4^ cells per well in a final volume of 100 μL of the culture medium. These cells were treated for 24 h with different *Premna odorata* extracts (groups 3–5) and silymarin (group 6) using 10 µL at 37 °C with 5% CO_2_. After the treatment, the cells were immediately incubated for 24 h at 37 °C [56]. The different groups were then treated with 10 mM 2’,7’-dichlorodihydrofluorescein diacetate (H_2_DCF–DA) dissolved in the phosphate buffer saline (PBS) (5.0 mg/mL) at 37 °C for 20 min. The color reaction was stopped by removing the media and adding 100 μL DMSO in each well to dissolve the formed formazan crystals. The incubation at 37 °C resumed for up to 20 min to ensure complete dissolution of crystals. The absorbance was determined at λ_495_ nm using an FLX800 fluorescence microplate reader (BioTEK Instruments, Winooski, VT, USA). H_2_O_2_ was used as the positive control.

### 4.6. Animal Treatment

Female Wistar albino rats (150–170 g) were obtained from the animal house of the National Research Centre, Dokki, Giza, Egypt. The rats were fed using a standard diet and free access to tap water [57]. The rats were housed in polypropylene cages and maintained under controlled conditions of the 12 h of light/12 h of dark cycle with 50% relative humidity at 25 to 30 °C with keeping for two weeks to be acclimatized to the environmental conditions.

### 4.7. Animal Ethical Statement

This study was approved by the Institutional Animal Ethics Committee of the National Research Center which stated that animals should not suffer at any stage of experimentation and be maintained in accordance with the Guide for the care and use of laboratory animals (ethical approval No: 012234).

### 4.8. Acute Toxicity Test

Acute toxicity studies were carried out using female Wistar albino rats as per Organization for Economic Cooperation and Development (OECD) guideline 423 (2001). According to the latter, acute oral toxicity refers to the adverse effects occurring as a result of oral administration of a single dose of a substance or multiple doses given within 24 h (overnight). Fasted rats were weighed and divided randomly into five groups containing three rats each. As there was no information regarding the plant being tested, for animal welfare reasons, the OECD recommends using a starting dose of 300 mg/kg b.w. If mortality was not observed after 24 h, the previous procedure was repeated for further groups with higher doses: 1000, 2000, 3000, 4000, and 5000 mg/kg b.w.

### 4.9. Induction of Alcohol Liver Inflammation and Experimental Design

Acute alcohol liver inflammation was induced in rats according to Keegan (2013) [58]. In a brief manner, rats received 2% sucrose as the sole source of liquid for three days prior to the initiation of 5% ethanol in 2% sucrose. The ethanol concentration was increased in increments of 5% at intervals of four days to a concentration of 15%. Thereafter, weekly increases were made to a final concentration of 40%. Fresh drinking water/ethanol was provided twice weekly. Sixty female Wistar albino rats were divided into six groups each containing ten rats: group 1: healthy rats (negative control); group 2: untreated rats (positive control) euthanized after thirty days; groups 3–5: rats treated orally through gavages with crude, defatted crude and n-hexane extracts, respectively, in the dose of 500 mg/kg b.w. for thirty days and then euthanized; group 6: rats treated orally through gavages with the silymarin reference drug [59] using the 200 mg/kg b.w. dose for thirty days and then euthanized.

#### 4.9.1. Blood Sampling

The blood samples were collected in a clean and dry test tube by puncturing of the sublingual vein. Subsequently, allowing the clotting process to last 10 min, they were centrifuged at 108,669× *g* for serum separation. The serum was stored at −80 °C for further experiments.

#### 4.9.2. Biochemical Analysis

The separated serum was used for the following tests: liver function tests: bilirubin was determined according to Walter and Gerade (1970) [60]; AST, ALT were determined according to Reitman and Frankel (1957) [61]; ALP was determined according to Belfield and Goldberg (1971) [62] using bio-diagnostic kits (Egypt); oxidative stress marker and antioxidant tests: MDA was determined according to Santos et al. (1980) [63]; GSH was determined according to Kageyama (1971) [64], and the TAC was determined according to Mclemore et al. (1998) [65]; inflammatory marker and adhesion molecule tests: CRP, TNF-α were determined according to Erhardt et al. (2004) [66] and Perrey et al. (1999) [67], respectively; ICAM-1 and VCAM-1 were determined according to Szarka et al. (2010) [68].

### 4.10. Statistical Analysis

The pooled data were presented as the means ± standard deviation (SD) for ten rats in each group. The differences between different treatment groups were determined by the ANOVA followed by the Dunnett’s test using PASW Statistics^®^ version 18 (Quarry Bay, Hong Kong, China), where an unshared letter is considered significant at *p* ≤ 0.05.

(1) $$\%\mathrm{change}=\frac{mean\;of\;negative\;control-mean\;of\;treatment\;group}{mean\;of\;negative\;control}\times 100$$

(2) $$\%\mathrm{improvment}=\frac{mean\;of\;positive\;control-mean\;of\;treatment\;group}{mean\;of\;negative\;control}\times 100$$

### 4.11. Histopathological Examination

The histological sections of the liver for all experimental groups of rats were taken by adopting the procedure described by Gomori (1941) [69]. Briefly, liver slices were taken from all groups and fixed instantaneously in neutral buffered formalin (10%) for 24 h, then processed in automatic processors, embedded in paraffin wax (melting point 55–41 °C), and paraffin blocks were obtained. Sections of 6 μm thickness were prepared and stained with the hematoxylin and eosin (H&E) stain. The cytoplasm stained shades of pink and red, while the nuclei gave a blue color. The slides were examined and photographed under a light microscope at the ×150 magnification power.

## 5. Conclusions

To sum up, our results showed 28 metabolites related to iridoids and polyphenolics; they were dereplicated and their biological relevance was correlated. The crude extract showed better activity in normalizing most of the parameters, indicating its capability to improve the inflamed liver in vitro and in vivo through its antioxidant capabilities, which was clear in all the chemical and histological examinations. The in silico ADMET screening study showed great bioavailability and distribution of different components. These findings support the use of these extracts due to the combined effects of these phytochemicals and/or their synergistic interactions as a natural remedy to improve the inflamed liver function.

## Figures and Tables

**Table 1:** The liquid chromatography/high-resolution electrospray ionization mass spectroscopy (LC–HRESIMS) dereplication results of the crude *Premna odorata* leave extract and fractions (n-hexane, dichloromethane, ethyl acetate, n-butanol).

No.	Identified	Source	MF	t_R_(min.)	*m/z*	Adduct	CE	H	DCM	EtOAC	B
**1**	Vitexin	*Premna odorata*	C_21_H_20_O_10_	9.71	433.1361	[M + H]^+^	+				+
**2**	Premnoside A	*Premna odorata*	C_39_H_44_O_20_	11.37	833.2746	[M + H]^+^	+			+	+
**3**	6- O-α- L-(2’’-O-trans-caffoyl) rhamnopyranosyl catalpol	*Premna odorata*	C_30_H_38_O_17_	11.98	671.1910	[M + H]^+^	+			+	+
**4**	Daucosterol	*Premna japonica*	C_35_H_60_O_6_	12.33	577.1969	[M + H]^+^	+	+			
**5**	Premnoside D	*Premna odorata*	C_39_H_44_O_19_	12.61	817.2282	[M + H]^+^	+			+	+
**6**	Premnoside H	*Premna odorata*	C_39_H_44_O_18_	13.00	801.2404	[M + H]^+^	+			+	+
**7**	Premnoside C	*Premna odorata*	C_40_H_46_O_19_	13.09	831.2411	[M + H]^+^	+			+	+
**8**	6- O-α- L-(2’’-O-trans-p-methoxycinnamoyl) rhamnopyranosyl catalpol	*Premna japonica*	C_31_H_40_O_16_	13.20	669.1634	[M + H]^+^	+			+	+
**9**	Stigmasterol	*Premna odorata*	C_29_H_48_O	13.33	413.2619	[M + H]^+^	+	+			
**10**	Acacetin	*Premna odorata*	C_16_H_12_O_5_	13.60	285.1126	[M + H]^+^	+			+	
**11**	Premnoside G	*Premna odorata*	C_39_H_44_O_18_	13.72	801.2404	[M + H]^+^	+			+	+
**12**	6- O-α- L-(4’’-O-trans-feruloyl) rhamnopyranosyl catalpol	*Premna japonica*	C_31_H_40_O_17_	13.78	685.2780	[M + H]^+^	+			+	+
**13**	β-sitosterol	*Premna odorata*	C_29_H_50_O	14.19	414.1849	[M + H]^+^	+	+			
**14**	Verbascoside	*Premna odorata*	C_29_H_36_O_15_	14.47	625.1396	[M + H]^+^	+			+	+
**15**	Premnoside F	*Premna odorata*	C_41_H_48_O_18_	14.54	829.2011	[M + H]^+^	+			+	+
**16**	Premnoside E	*Premna odorata*	C_41_H_48_O_18_	14.73	829.2011	[M + H]^+^	+			+	+
**17**	Linolenic acid	*Premna microphylla*	C_18_H_30_O_2_	14.81	277.1807	[M - H]^+^	+	+			
**18**	Diosmetin	*Premna odorata*	C_16_H_12_O_6_	15.09	301.2947	[M + H]^+^	+		+	+	
**19**	Premnaodoroside A	*Premna odorata*	C_42_H_66_O_20_	15.94	891.3561	[M + H]^+^	+		+		
**20**	Premnaodoroside B	*Premna odorata*	C_42_H_66_O_19_	16.06	875.2437	[M + H]^+^	+		+		
**21**	Premnaodoroside C	*Premna odorata*	C_42_H_64_O_19_	16.12	873.3192	[M + H]^+^	+		+		
**22**	Premnoside D	*Premna odorata*	C_40_H_46_O_20_	16.21	847.2782	[M + H]^+^	+			+	+
**23**	Premnaodoroside D	*Premna subscandens*	C_42_H_64_O_20_	17.74	889.2297	[M + H]^+^	+		+		
**24**	Β-amyrin	*Premna odorata*	C_30_H_50_O	18.28	465.2018	[M + K]^+^	+	+			
**25**	Luteolin	*Premna odorata*	C_15_H_10_O_6_	18.66	309.2349	[M + Na]^+^	+		+	+	
**26**	Apigenin	*Premna odorata*	C_15_H_10_O_5_	20.82	293.2147	[M + Na]^+^	+			+	
**27**	Premcoryoside	*Premna corymbosa*	C_45_H_58_O_24_	21.52	983.4891	[M + H]^+^	+				+
**28**	Arjunolic acid	*Premna microphylla*	C_30_H_48_O_5_	21.64	489.2793	[M + H}^+^	+	+			

MF: molecular formula, t_R_: retention time, min.: minute, CE: crude extract, H: n-hexane fraction, DCM: dichloromethane fraction, EtOAC: ethyl acetate fraction, B: n-butanol fraction.

**Table 2:** The predicted absorbance, distribution, metabolism, excretion (ADME) properties of the LC–HRESIMS dereplication metabolites of the crude *Premna odorata* leave extract using the in silico predicts absorption, distribution, metabolism, excretion, and toxicity (PreADMET) method.

No.	PPB%	BBB (C_brain_/C_blood_)	SP (cm/hour)	HIA%	MDCK (nm/sec)	Caco-2 (nm/sec)	Pgp Inhibition	CYP-_2_C_19_ Inhibition	CYP-_2_C_9_ Inhibition	CYP-_3_A_4_ Inhibition	CYP-_3_A_4_ Substrate
**1**	61.323656	0.0385273	−4.61128	31.374153	0.5424090	5.48785	No	Inhibitor	Inhibitor	Inhibitor	Weak
**2**	54.348583	0.0287162	−3.02912	3.7348170	0.0447556	13.6259	Inhibitor	Inhibitor	Inhibitor	Inhibitor	Weak
**3**	33.285061	0.0298880	−4.70662	3.3153240	0.1538230	11.0644	Inhibitor	Inhibitor	Inhibitor	Inhibitor	Weak
**4**	100.000000	5.3038700	−2.20420	90.027561	0.1220710	25.2333	Inhibitor	No	Inhibitor	Inhibitor	Substrate
**5**	54.577268	0.0305549	−2.97228	8.4947690	0.0455697	14.1947	Inhibitor	Inhibitor	Inhibitor	Inhibitor	Weak
**6**	56.739888	0.0342009	−2.91786	19.057599	0.0454732	14.7567	Inhibitor	Inhibitor	Inhibitor	Inhibitor	Substrate
**7**	48.364678	0.0424033	−2.96634	16.156072	0.0454091	14.0366	Inhibitor	Inhibitor	Inhibitor	Inhibitor	Weak
**8**	33.832287	0.06277950	−4.6442	13.170559	0.1289870	12.3956	Inhibitor	Inhibitor	Inhibitor	Inhibitor	Weak
**9**	100.000000	19.8938000	−0.717667	100.00000	3.783450	52.3376	Inhibitor	No	Inhibitor	Inhibitor	Substrate
**10**	90.917451	0.15030900	−3.36001	93.042708	20.230800	12.7923	No	Inhibitor	Inhibitor	Inhibitor	No
**11**	53.912360	0.1319930	−2.9222	52.345314	0.0449026	15.3124	Inhibitor	Inhibitor	Inhibitor	Inhibitor	Weak
**12**	32.612262	0.0411953	−4.70477	5.9983830	0.1105100	9.11617	Inhibitor	Inhibitor	Inhibitor	Inhibitor	Weak
**13**	100.000000	19.8883000	−0.593439	100.00000	8.8571900	52.3734	Inhibitor	No	Inhibitor	Inhibitor	Substrate
**14**	64.288492	0.03167600	−3.5116	7.6711810	0.0450549	11.1087	Inhibitor	Inhibitor	Inhibitor	Inhibitor	Weak
**15**	54.629780	0.1252660	−2.92245	52.345571	0.0451432	15.4511	Inhibitor	Inhibitor	Inhibitor	Inhibitor	Weak
**16**	53.912360	0.1319930	−2.9222	52.345314	0.0449026	15.3124	Inhibitor	Inhibitor	Inhibitor	Inhibitor	Weak
**17**	100.000000	6.16921000	−0.538273	98.273607	74.789700	27.9738	Inhibitor	Inhibitor	Inhibitor	Inhibitor	No
**18**	90.160128	0.20108600	−4.13473	88.188263	23.853100	7.02526	No	Inhibitor	Inhibitor	Inhibitor	No
**19**	34.413157	0.0284513	−3.29001	1.9433930	0.2328650	16.3835	Inhibitor	Inhibitor	Inhibitor	Inhibitor	Substrate
**20**	40.461576	0.0298628	−3.70182	4.3580790	0.0841395	17.2768	Inhibitor	Inhibitor	Inhibitor	Inhibitor	Substrate
**21**	41.088270	0.0302347	−3.26196	5.1422870	0.1599090	15.3574	Inhibitor	Inhibitor	Inhibitor	Inhibitor	Substrate
**22**	45.368220	0.0328157	−3.02594	7.1270550	0.0447377	12.7477	Inhibitor	Inhibitor	Inhibitor	Inhibitor	Weak
**23**	34.183542	0.0283962	−3.80436	2.2958830	0.291627	17.4161	Inhibitor	Inhibitor	Inhibitor	Inhibitor	Substrate
**24**	100.000000	21.2500000	−2.22251	100.00000	0.1749200	46.7500	Inhibitor	No	Inhibitor	Inhibitor	Substrate
**25**	99.717233	0.36758200	−4.28017	79.427233	36.520500	4.53973	No	Inhibitor	Inhibitor	Inhibitor	No
**26**	97.253409	0.56511300	−4.14570	88.122839	44.302000	10.5468	No	Inhibitor	Inhibitor	Inhibitor	No
**27**	37.405289	0.0273879	−2.50822	0.3453560	0.0434853	11.7965	Inhibitor	Inhibitor	Inhibitor	Inhibitor	Substrate
**28**	97.049829	0.58860800	−3.57106	91.233319	0.0434480	20.9815	Inhibitor	No	Inhibitor	Inhibitor	Substrate

PPB: plasma protein binding; BBB: blood–brain barrier; SP: skin permeability; HIA: human intestinal absorption; MDCK: Madin–Darby Canine Kidney; Pgp: permeability glycoprotein; and CYP: cytochrome P. In PPB, the drug is considered; chemicals are strongly PPB at ˃ 90% PPB and weakly at ˂ 90%. In the BBB, the drug is considered to have high absorption to the CNS at ˃ 2.0 BB (Cbrain/Cblood), middle absorption to the CNS at 2.0~0.1 BB (Cbrain/Cblood), and low absorption to the CNS at ˂ 0.1 BB (Cbrain/Cblood). For SP, PreADMET predicts in vitro skin permeability and the result value is given as logKp. Kp (cm/hour) is defined as follows: KP =$\;\frac{\mathrm{km}\times D}{h}$, where *Km* is the coefficient of distribution between the stratum corneum and the vehicle, *D* is the average diffusion coefficient (cm^2^/h), and *h* is the thickness of skin (cm). In HIA, the drug is considered a poorly absorbed compound at 0~20%, a moderately absorbed compound at 20~70%, and a well-absorbed compound at 70~100%. In the Caco-2 cell permeability and the MDCK cell model, the drug could generally belong to one of three categories: low permeability ˂ 4 P_Caco−2_ (nm/sec), moderate permeability 4~70 P_Caco−2_ (nm/sec), and high permeability ˃ 70 P_Caco−2_ (nm/sec). Pgp is an important protein of the cell membrane that pumps many foreign substances out of cells, and it likely evolved as a defense mechanism against harmful substances. Cytochrome P_450_-2C_19_ (CYP_2_C_19_), CYP_2_C_9_, and CYP_3_A_4_, are important cytochrome P_450_ enzymes with a major role in the oxidation of both xenobiotic and endogenous compounds.

**Table 3:** Toxicity profile of the LC–HRESIMS dereplication metabolites of *Premna odorata* leaves crude extract using in silico predicts absorption, distribution, metabolism, excretion, and toxicity (PreADMET) method.

No.	Ames Test	TA100-10RLI	TA100-NA	TA1535-10RLI	TA1535-NA	Carcinogenic for Mice	Carcinogenic for Rats	HERG Inhibition
**1**	Non-mutagenic	−	−	−	−	+	−	High risk
**2**	Non-mutagenic	−	−	−	−	+	−	Ambiguous
**3**	Non-mutagenic	−	−	−	−	+	−	Ambiguous
**4**	Non-mutagenic	−	−	−	−	+	−	Low risk
**5**	Non-mutagenic	−	−	−	−	+	−	Ambiguous
**6**	Non-mutagenic	−	−	−	−	+	−	Ambiguous
**7**	Non-mutagenic	−	−	−	−	+	−	Ambiguous
**8**	Non-mutagenic	−	−	−	−	+	−	High risk
**9**	Non-mutagenic	−	−	−	−	+	+	Low risk
**10**	Mutagenic	+	+	−	−	+	+	Moderate risk
**11**	Non-mutagenic	−	−	−	−	+	−	Ambiguous
**12**	Non-mutagenic	−	−	−	−	+	−	Ambiguous
**13**	Non-mutagenic	−	−	−	−	+	−	Low risk
**14**	Non-mutagenic	−	−	−	−	+	−	Ambiguous
**15**	Non-mutagenic	−	−	−	−	+	−	High risk
**16**	Non-mutagenic	−	−	−	−	+	−	High risk
**17**	Mutagenic	−	−	−	+	+	+	Moderate risk
**18**	Mutagenic	−	+	−	−	+	+	Moderate risk
**19**	Mutagenic	−	+	−	−	+	−	Ambiguous
**20**	Non-mutagenic	−	−	−	−	+	−	Ambiguous
**21**	Mutagenic	−	+	−	−	+	−	Ambiguous
**22**	Non-mutagenic	−	−	−	−	+	−	Ambiguous
**23**	Mutagenic	−	+	−	−	+	−	Ambiguous
**24**	Non-mutagenic	−	−	−	−	+	+	Low risk
**25**	Mutagenic	−	+	−	−	+	+	Moderate risk
**26**	Mutagenic	+	+	−	−	+	+	Moderate risk
**27**	Non-mutagenic	−	−	−	−	+	−	Ambiguous
**28**	Non-mutagenic	−	−	−	−	+	+	Low risk

**Table 4:** Results of the liver function, oxidative stress marker, antioxidant, inflammatory marker, and adhesion molecule tests measuring the activity of the *Premna odorata* crude, defatted, and n-hex extracts induced in alcohol-inflamed liver female Wistar albino rats according to Keegan (2013) [23] using a dose of 500 mg/kg b.w. for thirty days who were then euthanized (silymarin was used as a reference drug using a 200 mg/kg b.w. dose).

		Groups					
Parameters		1	2	3	4	5	6
Bilirubin (mg/dl)	Mean ± SD	0.85 ± 0.05 ^b^	1.41 ± 0.18 ^a^	0.916 ± 0.07 ^b^	0.95 ± 0.05 ^b^	0.916 ± 0.076 ^b^	0.8 ± 0.05 ^b^
	% change		65.88	7.76	11.76	7.76	5.88
	% improvement			58.11	54.11	58.11	71.76
AST (U/I)	Mean ± SD	39.00 ± 7.81 ^b^	81.33 ± 21.45 ^a^	25.47 ± 4.50 ^d^	28.33 ± 1.53 ^cd^	32.33 ± 2.08 ^bc^	31.47 ± 3.51 ^bcd^
	% change		108.53	34.20	27.30	17.10	19.31
	% improvement			143.23	133.23	125.64	127.84
ALT (U/I)	Mean ± SD	108.47 ± 18.85 ^b^	144.33 ± 25.1 ^a^	77.33 ± 4.42 ^bc^	72.00 ± 2.00 ^bc^	92.47 ± 16.28 ^b^	93.33 ± 5.77 ^b^
	% change		33.05	28.71	33.62	14.75	13.96
	% improvement			61.76	66.68	47.81	47.02
ALP (IU/L)	Mean ± SD	173.33 ± 29.29 ^bc^	232.33 ± 2.51 ^a^	148.33 ± 10.40 ^bc^	181.47 ± 7.44 ^b^	185 ± 18.02 ^b^	141.47 ± 2.51 ^c^
	% change		34.04	14.42	4.69	6.73	18.27
	% improvement			48.47	29.32	27.29	52.42
MDA (mmol/l)	Mean ± SD	2818.85 ± 200.5 ^c^	4,029.85 ± 200.5 ^a^	2,026.33 ± 52.50 ^d^	2,121.47 ± 91.76 ^d^	2,248.00 ± 141.24 ^d^	3,250.47 ± 416.41 ^b^
	% change		42.97	28.11	24.73	20.22	15.33
	% improvement			71.07	67.21	63.20	27.60
GSH (mg/g tissue used)	Mean ± SD	412.86 ± 56.94 ^bc^	299.16 ± 54.98 ^d^	491.91 ± 27.56 ^c^	506.88 ± 86.32 ^b^	495.46 ± 28.99 ^ab^	411.29 ± 77.11 ^bc^
	% change		27.42	19.15	22.77	20.26	0.24
	% improvement			46.60	50.32	47.57	27.18
TAC (mmol/l)	Mean ± SD	0.26 ± 0.02 ^a^	0.16 ± 0.02 ^b^	0.26 ± 0.02 ^a^	0.26 ± 0.02 ^a^	0.27 ± 0.03 ^a^	0.29 ± 0.02 ^a^
	% change		38.46	0	0	3.84	11.53
	% improvement			38.46	38.46	42.30	50.00
CRP (ng/mL)	Mean ± SD	29.30 ± 2.01 ^b^	54.58 ± 2.18 ^a^	32.47 ± 2.51 ^b^	33.33 ± 1.52 ^b^	29.03 ± 2.74 ^b^	31.20 ± 1.38 ^b^
	% change		86.96	10.34	13.79	1.02	6.48
	% improvement			75.86	72.40	86.20	80.34
TNF-α (pg/mL)	Mean ± SD	39.33 ± 2.47 ^bc^	73.03 ± 2.45 ^a^	43.47 ± 2.76 ^bc^	33.47 ± 1.53 ^c^	41.47 ± 2.39 ^bc^	52.41 ± 1.45 ^b^
	% change		98.49	10.25	15.38	5.12	33.74
	% improvement			76.92	102.56	82.76	53.84
ICAM-1 (μg/mL)	Mean ± SD	5.53 ± 0.47 ^c^	11.81 ± 1.22 ^a^	7.46 ± 1.41 ^b^	6.83 ± 0.77 ^bc^	6.49 ± 0.41 ^bc^	6.43 ± 0.56 ^bc^
	% change		114.54	36.36	23.50	18.18	16.30
	% improvement			78.18	90.05	96.20	98.18
VCAM-1 (μg/mL)	Mean ± SD	2.92 ± 0.12 ^b^	4.46 ± 1.30 ^a^	3.40 ± 0.20 ^b^	3.29 ± 0.23 ^b^	3.22 ± 0.58 ^b^	2.96 ± 0.11 ^b^
	% change		51.72	17.24	13.79	13.79	3.45
	% improvement			37.93	41.37	41.37	51.72

Group 1: negative control group; group 2: alcohol-inflamed liver (positive control group); group 3: crude extract-treated group; group 4: defatted crude extract-treated group; group 5: n-hexane extract-treated group; group 6: silymarin-treated group; AST: aspartate aminotransferase; ALT: alanine aminotransferase; ALP: alkaline phosphatase; MDA: malondialdehyde; GSH: glutathione; TAC: total antioxidant capacity; CRP: C-reactive protein; TNF-α: tumor necrosis factor-α; ICAM-1: intercellular adhesion molecule-1; VCAM-1: vascular cell adhesion molecule-1.Pooled data presented as the means ± standard deviation (SD) for ten rats in each group. The differences between various treatment groups determined by the ANOVA followed by the Dunnett’s test using PASW Statistics^®^ version 18 (Quarry Bay, Hong Kong), ^a^–^d^: Means with different letters in the same row differs significantly (*p* ≤ 0.05), where an unshared letter is considered significant at *p* ≤ 0.05.

**Table 5:** Kit reagents used in the different experiments.

Experiment	Kit Reagents
**ROS**	2’,7’-dichlorodihydrofluorescein diacetate (H_2_DCF–DA), Roswell Park Memorial Institute (RPMI) 1640 medium, fetal calf serum, penicillin, and streptomycin
**Bilirubin**	sulfanilic acid, hydrochloric acid, dimethyl sulfoxide
**AST**	phosphate buffer pH 7.5 (100 mmol/L), aspartate (10 0mmol/L), α-ketoglutarate (2 mmol/L)
**ALT**	alanine 200 mmol/L, 2,4-dinitrophenyl hydrazine (1 mmol/L)
**ALP**	standard phenol (1.59 mmol/L), buffer pH 10 (50 mmol/L), phenyl phosphate (5 mmol/L), EDTA (100 mmol/L), 4-aminophenazone (50 mmol/L), potassium ferricyanide (200 mmol/L)
**MDA**	standard MDA (10 mmol/mL), thiobarbituric acid (25 mmol/L), detergent (3 mmol/L), stabilizer (15 mmol/L)
**GSH**	DTNB (1 mmol/L)
**TAC**	sulfuric acid, sodium phosphate, ammonium molybdate
**CRP**	capture antibody-coated microplate: one plate of 96 wells coated with a rabbit anti-rat CRP antibody detection antibody/enzyme conjugates (100 x): concentrated horseradish peroxidase (HRP) conjugated to a rabbit anti-rat CRP antibody containing stabilizers and preservative standard (10 x): rat serum with elevated levels of CRP, wash buffer: powdered phosphate-buffered saline (PBS) with 0.05% Tween-20, TMB substrate: solution containing 3, 3’, 5, 5’-tetramethylbenzidine (TMB) stop solution: diluted phosphoric acid
**TNF-α**	Rat TNF-α microplates – 96-well polystyrene microplates (12 strips of 8 wells) coated with a monoclonal antibody specific to rat TNF-α Rat TNF-α conjugate – 23 mL/vial of a polyclonal antibody against the rat TNF-α conjugated to horseradish peroxidase with preservatives Rat TNF-α standard – 1.5 ng/vial of the recombinant rat TNF-α in a buffered protein base with preservatives, lyophilized Rat TNF-α control – the recombinant rat TNF-α in a buffered protein base with preservatives. lyophilized The concentration range of the rat TNF-α after reconstitution. The assay value of the control should be within the range specified on the label, assay diluent RD1-41 – 12.5 mL/vial of the buffered protein base with preservatives, calibrator diluent RDS-17 – 21 mL/vial of the buffered protein base with preservatives, wash buffer concentrate – 50 mL/vial of a 25-fold concentrated solution of a buffered surfactant with preservatives, color reagent A – 12.5 mL/vial of the stabilized hydrogen peroxidase, color reagent B – 12.5 mL/vial of the stabilized chromogen (tetramethylbenzidine) Stop solution – 23 mL/vial of a diluted hydrochloric acid solution, plate covers – adhesive strips
**VCAM-1**	Pre-coated, ready-to-use 96-well strip plate, plate sealer for 96 wells, standard diluent, assay diluent A, assay diluent B, stop solution, standard, detection reagent A, detection reagent B, TMB substrate, wash buffer (30 x concentrate);
**ICAM-1**	Pre-coated 96-well strip microplate, wash buffer, stop solution, assay diluent(s), lyophilized standard, biotinylated detection antibody, streptavidin-conjugated HRP, TMB One-Step Substrate
